# Organotypic Brain Slice Cultures of Adult Transgenic P301S Mice—A Model for Tauopathy Studies

**DOI:** 10.1371/journal.pone.0045017

**Published:** 2012-09-11

**Authors:** Agneta Mewes, Heike Franke, David Singer

**Affiliations:** 1 Institute of Bioanalytical Chemistry, Center for Biotechnology and Biomedicine (BBZ), University of Leipzig, Leipzig, Germany; 2 Rudolf-Boehm-Institute of Pharmacology and Toxicology, University of Leipzig, Leipzig, Germany; The Mental Health Research Institute, University of Melbourne, Australia

## Abstract

**Background:**

Organotypic brain slice cultures represent an excellent compromise between single cell cultures and complete animal studies, in this way replacing and reducing the number of animal experiments. Organotypic brain slices are widely applied to model neuronal development and regeneration as well as neuronal pathology concerning stroke, epilepsy and Alzheimer’s disease (AD). AD is characterized by two protein alterations, namely tau hyperphosphorylation and excessive amyloid β deposition, both causing microglia and astrocyte activation. Deposits of hyperphosphorylated tau, called neurofibrillary tangles (NFTs), surrounded by activated glia are modeled in transgenic mice, e.g. the tauopathy model P301S.

**Methodology/Principal Findings:**

In this study we explore the benefits and limitations of organotypic brain slice cultures made of mature adult transgenic mice as a potential model system for the multifactorial phenotype of AD. First, neonatal (P1) and adult organotypic brain slice cultures from 7- to 10-month-old transgenic P301S mice have been compared with regard to vitality, which was monitored with the lactate dehydrogenase (LDH)- and the MTT (3-(4,5-Dimethylthiazol-2-yl)-2,5-diphenyltetrazolium bromide) assays over 15 days. Neonatal slices displayed a constant high vitality level, while the vitality of adult slice cultures decreased significantly upon cultivation. Various preparation and cultivation conditions were tested to augment the vitality of adult slices and improvements were achieved with a reduced slice thickness, a mild hypothermic cultivation temperature and a cultivation CO_2_ concentration of 5%. Furthermore, we present a substantial immunohistochemical characterization analyzing the morphology of neurons, astrocytes and microglia in comparison to neonatal tissue.

**Conclusion/Significance:**

Until now only adolescent animals with a maximum age of two months have been used to prepare organotypic brain slices. The current study provides evidence that adult organotypic brain slice cultures from 7- to 10-month-old mice independently of the transgenic modification undergo slow programmed cell death, caused by a dysfunction of the neuronal repair systems.

## Introduction

The *ex vivo* culture system of organotypic brain slices was first introduced by Gähwiler establishing the roller-tube method [1]. After embedding in a plasma clot, the slices are cultivated on glass coverslips in a slowly rotating tube. The slices thin to a quasi-monolayer upon cultivation, revealing optimal properties for experiments that require single neuron access or optimal optic conditions. A second method for brain slice cultivation using semiporous membranes was established by Stoppini and coworkers [2]. In this model the three-dimensional tissue structure is well preserved, enabling morphological, biochemical and electrophysiological studies. Both methods have been applied to prepare organotypic brain slice cultures from neonatal rodents and the technique has become a sophisticated tool in neuroscience. These systems were used to explore the functional [3] and cellular development of the brain [4], [5] as well as in drug screenings for e.g. neuroprotective substances [6], [7]. Furthermore, organotypic brain slice cultures are widely applied to study biochemical changes related to lesions associated with stroke [8], epilepsy [9], and Alzheimer’s disease (AD) [10], [11], respectively. Especially in the case of AD research brain slices are favorable tools, because single cell cultures do not resemble the complex AD pathology. AD brain pathology is characterized by the extracellular deposition of amyloid β 1–42 (Aβ_1–42_) in amyloid plaques and the intracellular aggregation of hyperphosphorylated tau protein in neurofibrillary tangles (NFTs), all embedded in an environment of activated glial cells [12], [13], [14]. Several groups developed organotypic brain slice systems to model an AD like phenotype comprising Aβ deposits [11], [15] or tau hyperphosphorylation [10]. Among others, the AD mimicking pathology was introduced with ocadaic acid to trigger tau hyperphosphorylation [10] or biolistic transfection with amyloid precursor protein expression vectors [15]. However, these systems rely on neonatal tissue, which is characterized by a high resistance to ischemic damage [16], an immature metabolism state [17], immature astrocytes [18], an increased synaptic plasticity [19], and a complex pattern of dendritic branching [20]. These profound differences between neonatal and mature adult brain tissue influence experiments, which are related to age dependent changes like those found in AD. Several attempts to cultivate 20- to 30-day-old adolescent brain tissue for one to twelve weeks have been reported [3], [21], [22] as well as the preparation of two months old rodents [23], [24]. The oldest rodents used for organotypic tissue cultures of the hippocampus were 14–16 months old [25]. Organotypic brain slice models of mature transgenic mice, which develop an AD-like pathology, would benefit from mature adult brain morphology, metabolism and, for example, neuronal tau pathology surrounded by activated glia.

This study evaluates an organotypic brain slice model of adult transgenic P301S mice [26]. Heterozygous animals *in vivo* show the first pathological alternation at three months of age, i.e. brain atrophy, microglia activation, and tau-positive spheroids in the cerebral cortex. At six months of age the tau pathology is fully developed, displaying NFTs in neocortex, amygdala, hippocampus, brain stem, and spinal cord associated with a decreased solubility of the tau protein, impaired synaptic transmission and astrogliosis. Later on hippocampal neuronal loss (8 months) and a strong paralysis (7–10 months) are observed [26]. Our study relied on 7- to 10-month-old mature adult P301S mice with established tau pathology for organotypic brain slice experiments and examined various preparation and cultivation conditions for these adult brain slice cultures. Compared to neonatal organotypic brain slices, we investigated cell vitality and characterized alterations in cellular morphology.

## Results

### Vitality of Neonatal and Adult Organotypic Brain Slices

**Figure 1:**
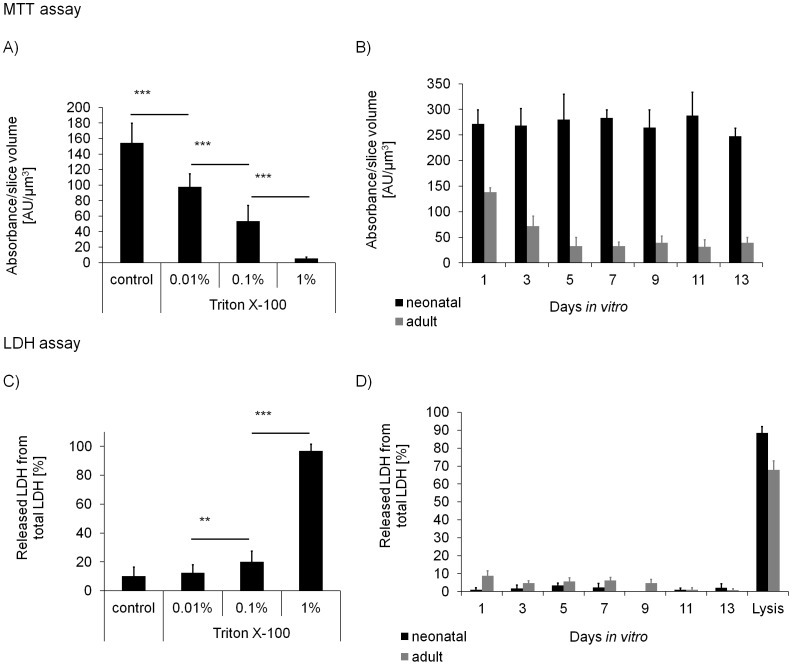
Metabolism rate and cellular necrosis in neonatal and adult organotypic brain slice cultures. A and C: Reliability of MTT and LDH assays shown by a concentration dependent Triton X-100 induced cell death in adult organotypic brain slice cultures. B and D: Cellular metabolism rate (B) and cellular necrosis (D) in neonatal and adult organotypic brain slice cultures. n = 12 (MTT assay), n = 9 (LDH assay), ***p<0.001, **p<0.01.

LDH and MTT assays were used to determine the vitality of the cultivated brain slices. The first assay quantifies LDH released from cells with damaged cellular membranes, which is indicative for the number of necrotic cells [27], [28]. The second assay is based on the conversion of yellow MTT to purple crystalline formazan by metabolic active cells [29]. Both assays were suitable to monitor Triton X-100 induced necrosis in a concentration dependent manner. The MTT assay (Fig. 1A) was more sensitive than the LDH assay (Fig. 1C), significantly (p<0.001) distinguishing control slices from slices treated with 0.01% Triton X-100. The metabolism rate of neonatal brain slices was stable at an average level of 250 AU/µm^3^ over a cultivation period of 15 days (Fig. 1B). In contrast, the metabolism rate of adult brain slices was 140 AU/µm^3^, i.e. approximately 50% lower at the 1^st^ day *in vitro* (DIV). The metabolism rate decreased further to 70 AU/µm^3^ at the 3^rd^ DIV. From the 5^th^ DIV on to the 15^th^ DIV the metabolism rate remained stable at a level of approximately 50 AU/µm^3^, i.e. around 35% of the original activity. The LDH assay revealed a slightly different vitality level between neonatal and adult brain tissue, with approximately 90% cells being alive at the 15^th^ DIV in the neonatal slice cultures and an average of 70% cells in the adult slice cultures (Fig. 1D). Over the first 9 days of cultivation, adult slices showed a 3–8% higher LDH release compared to neonatal slices. After the 11^th^ DIV the LDH efflux was approximately 0–3% for both, neonatal and adult brain slices. Importantly, there was no significant vitality difference of brain slices from transgenic P301S mice compared to wild type mice (Fig S1).

### Optimization of Preparation and Cultivation Conditions for Adult Organotypic Brain Slices

When the preparation and cultivation conditions of neonatal slice cultures were applied to adult slice cultures, both vitality assays revealed a significant difference between neonatal and adult slices (Fig. 1B, 1D). To improve the vitality of adult brain slice cultures, we investigated alternative preparation and cultivation procedures. The vitality of the tissue cultures was again assessed by the MTT assay (table 1) and the LDH assay (table 2). Perfusion with ice-cold preparation buffer for a rapid tissue cooling compared to direct decapitation did not affect the slice vitality. In contrast, the reduction of the slice thickness from 300 to 200 µm resulted in a 30–50% higher metabolism rate and a 5–10% lower LDH release by providing a better nutrition supply. An increase in slice thickness (400 µm) appeared to reduce the vitality of the slice cultures, but this effect was not significant. As a reduced cultivation temperature possibly decrease ischemic processes in the tissue cultures [30], we analyzed brain slices cultured at 32°C and 35°C. Both conditions resulted in comparable metabolic states, but slices cultured at 32°C showed a 50% higher LDH release. Different CO_2_ concentrations were tested to minimize pH variations [31] but slices cultivated at 5% CO_2_ rather than 2.5% CO_2_ demonstrated a 30–40% higher metabolism rate and a 20% lower LDH release. Starting from a standard medium composed of DMEM Ham’s F12 and 24% normal horse serum, containing antibiotics and D-glucose, a series of additives was analyzed to probably aid the cell survival. The addition of lactate and β-hydroxybutyrate as alternative energy sources [32] did not improve the tissue vitality. The exchange of normal horse serum to the B27 supplement, a synthetic substitute for serum to minimize batch to batch variations and glial scaring, had no influence on the metabolism rate, but these slices displayed a 25% elevated LDH release. Furthermore, increasing the potassium concentration from 4 to 10 mM [33] did not affect the vitality of the slice cultures. The addition of survival promoting peptides (SPPs) and additives like MK-801 or antisense oligodesoxynucleotides (AsODN) against connexin 43 and butylated hydroxytoluene (BHT), which were shown to be beneficial for cell survival in adolescent brain slice cultures [23], [34]–[36], did also not alter the viability of mature adult brain slices.

**Table 1:** Quantification of the metabolism rate (MTT assay) over a period of 15 days under various preparation and cultivation conditions for adult organotypic brain slice cultures*.

	Days *in vitro*	1	3	5	7	9	11	13	15
Mode of preparation	Direct decapitation	167±25	95±26	80±17	66±13	72±21	68±23	48±10	51±10
	Perfusion	147±28	74±22	62±17	69±21	61±15	52±9	44±10	48±10
Slice thickness	200 µm	138±8	72±19	33±17	33±8	39±13	32±14	39±10	27±11
	300 µm	70±27	30±9	21±6	15±6	11±3	13±4	17±6	20±8
	400 µm	62±16	26±20	17±6	13±5	10±5	10±3	11±6	12±3
Temperature	35°C	141±13	79±17	50±12	63±17	38±13	48±11	35±9	45±11
	32°C	133±28	80±16	65±21	53±21	34±14	36±14	33±9	42±8
CO_2_ concentration	5%	150±24	93±10	63±14	60±14	70±17	51±8	62±13	63±18
	2.5%	143±26	79±19	44±13	34±14	53±11	34±10	34±9	41±14
Media	With glucose and NHS	142±36	89±25	61±18	65±12	36±15	42±20	51±7	45±14
	With substrate mix and NHS	146±22	76±12	66±30	42±11	35±11	38±9	33±13	17±7
	With glucose and B27 supplement	147±32	86±19	71±21	42±25	35±11	28±14	21±11	16±5
Potassium concentration	4 mM	146±21	65±14	62±16	59±15	63±21	54±20	47±10	50±14
	10 mM	139±19	62±11	46±21	50±9	51±12	48±9	53±13	47±12
Survival promoting peptides	Without	197±35	87±13	64±18	60±20	48±7	38±14	39±13	46±9
	SPP 1	183±24	87±13	36±22	46±15	43±18	39±10	32±20	37±22
	SPP 2	168±31	73±15	49±16	48±8	31±15	46±11	50±13	58±13
	SPP 3	183±40	86±12	64±27	49±26	50±25	50±12	61±13	60±17
Additives	Without	201±44	82±16	71±16	62±14	41±10	55±14	43±8	46±4
	BHT	122±38	70±28	69±21	52±20	37±20	35±13	34±7	33±11
	AsODN against connexin 43	140±40	70±15	46±4	58±8	39±16	40±13	42±10	30±6
	MK-801	177±21	99±14	75±12	76±13	56±20	39±13	45±9	49±13

* Values are given as absorbance (562 nm–690 nm) per slice volume [AU/µm^3^] ± standard deviation. n = 7–8 (NHS: normal horse serum; SPP: survival promoting peptide; BHT: butylated hydroxytoluene; AsODN: antisense oligodesoxynucleotides).

**Table 2:** Quantification of the LDH release over a period of 15 days under various preparation and cultivation conditions for adult organotypic brain slice cultures*.

	Days *in vitro*	1	3	5	7	9	11	13	Lysis
Mode of preparation	Direct decapitation	8±3	6±2	4±1	4±1	4±1	3±1	2±1	65±6
	Perfusion	9±2	8±3	6±3	7±2	5±1	4±1	3±0	58±9
Slice thickness	200 µm	4±4	1±1	1±1	1±1	1±1	1±0	3±3	88±5
	300 µm	6±6	3±3	3±4	1±1	1±1	3±3	5±5	78±13
	400 µm	8±5	9±10	4±3	3±2	2±2	2±1	2±1	71±13
Temperature	35°C	2±1	1±1	2±1	8±2	21±5	1±1	3±1	63±5
	32°C	14±4	14±2	15±3	8±4	14±3	1±1	0±0	34±10
CO_2_ concentration	5%	12±5	3±3	3±3	3±2	4±1	4±1	3±1	67±11
	2.5%	10±7	6±5	7±4	9±3	6±3	5±3	3±2	54±15
Media	With glucose and NHS	9±3	5±1	6±2	6±2	5±2	1±1	1±1	68±5
	With substrate mix and NHS	10±3	6±2	4±4	6±3	4±2	0±1	1±1	69±11
	With glucose and B27 supplement	9±6	11±4	25±5	2±3	0±1	0±1	1±1	52±9
Potassium concentration	4 mM	8±1	6±1	5±1	3±1	0±0	0±0	0±0	77±3
	10 mM	10±3	8±2	6±1	2±1	0±0	0±0	0±0	73±6
Survival promoting peptides	Without	13±2	3±3	4±5	0±0	1±1	2±2	2±2	75±5
	SPP 1	14±3	1±1	7±7	1±2	2±1	2±2	2±2	72±7
	SPP 2	14±3	1±2	5±5	0±0	2±2	1±1	2±2	75±4
	SPP 3	14±6	1±2	3±5	0±0	1±1	1±1	3±2	76±6
Additives	Without	10±7	15±7	12±6	4±2	0±0	0±0	0±1	60±7
	BHT	24±3	0±0	0±0	0±1	2±2	2±2	9±5	63±5
	AsODN against connexin 43	26±4	16±4	9±5	4±4	0±0	0±0	3±7	43±7
	MK-801	22±4	15±3	11±2	4±3	2±2	0±0	0±0	46±7

* Values are given as percentage of the released total LDH ± standard deviation. n = 11–12 (NHS: normal horse serum; SPP: survival promoting peptide; BHT: butylated hydroxytoluene; AsODN: antisense oligodesoxynucleotides).

### Morphological Characterization of Organotypic Brain Slices

#### Directly fixed neonatal and adult brain tissue

Cortical neurons were structured in six distinct layers in neonatal as well as adult brain slices. Neurons differed in shape and size due to their cortical localization. Astrocytes were rarely present in healthy neonatal and adult cortical structures and found as single cells or small clusters (Fig. S2 A, B, C). Additionally, only neonatal tissue presented the characteristic radial glial cells. Microglia cells occurred in the same amount in neonatal and adult brain tissue (Fig. S3 A, B, C). In healthy intact tissue the microglial cells were in a ramified state, comprising small cell bodies with long and thin branches.

**Figure 2:**
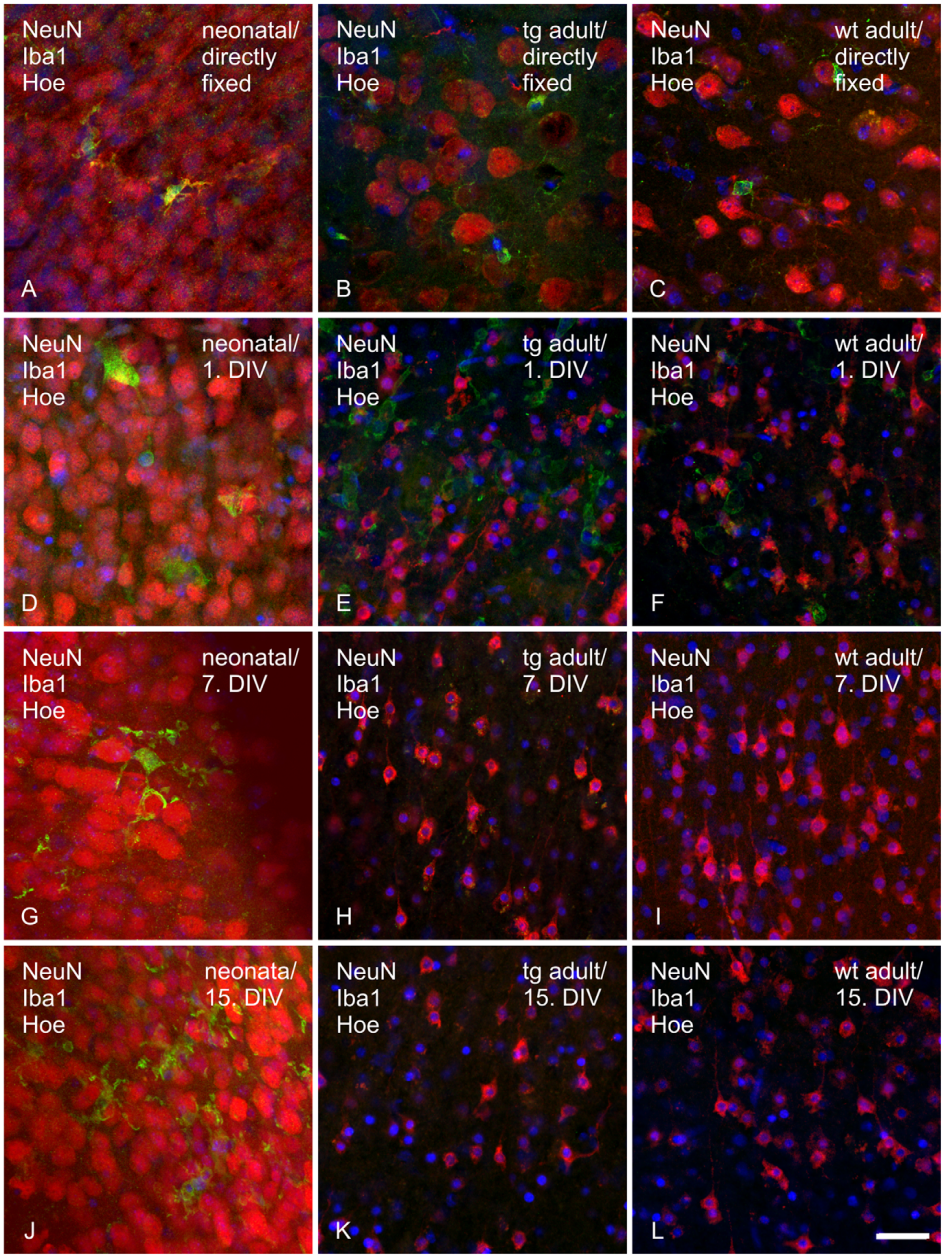
Immunohistochemical labeling of neurons and microglia in directly fixed and cultivated brain tissue. Neurons (red), microglia (green) and cell nuclei (blue) were visualized with anti-NeuN antibody, anti-Iba1 antibody and Hoechst, respectively, in the somatosensory cortex (layer VI) of directly fixed (30 µm) and cultivated brain tissue of neonatal (wt/tg mixed, 300 µm) and adult wild type and transgenic P301S mice (200 µm). A–C: directly fixed; D–F: fixed on 1^st^ DIV; G–I: fixed on 7^th^ DIV; J–L: fixed on 15^th^ DIV. A, D, G, J: neonatal tissue; B, E, H, K: transgenic (tg) adult tissue; C, F, I, L: wild type (wt) adult tissue. Scale bar 20 µm.

**Figure 3:**
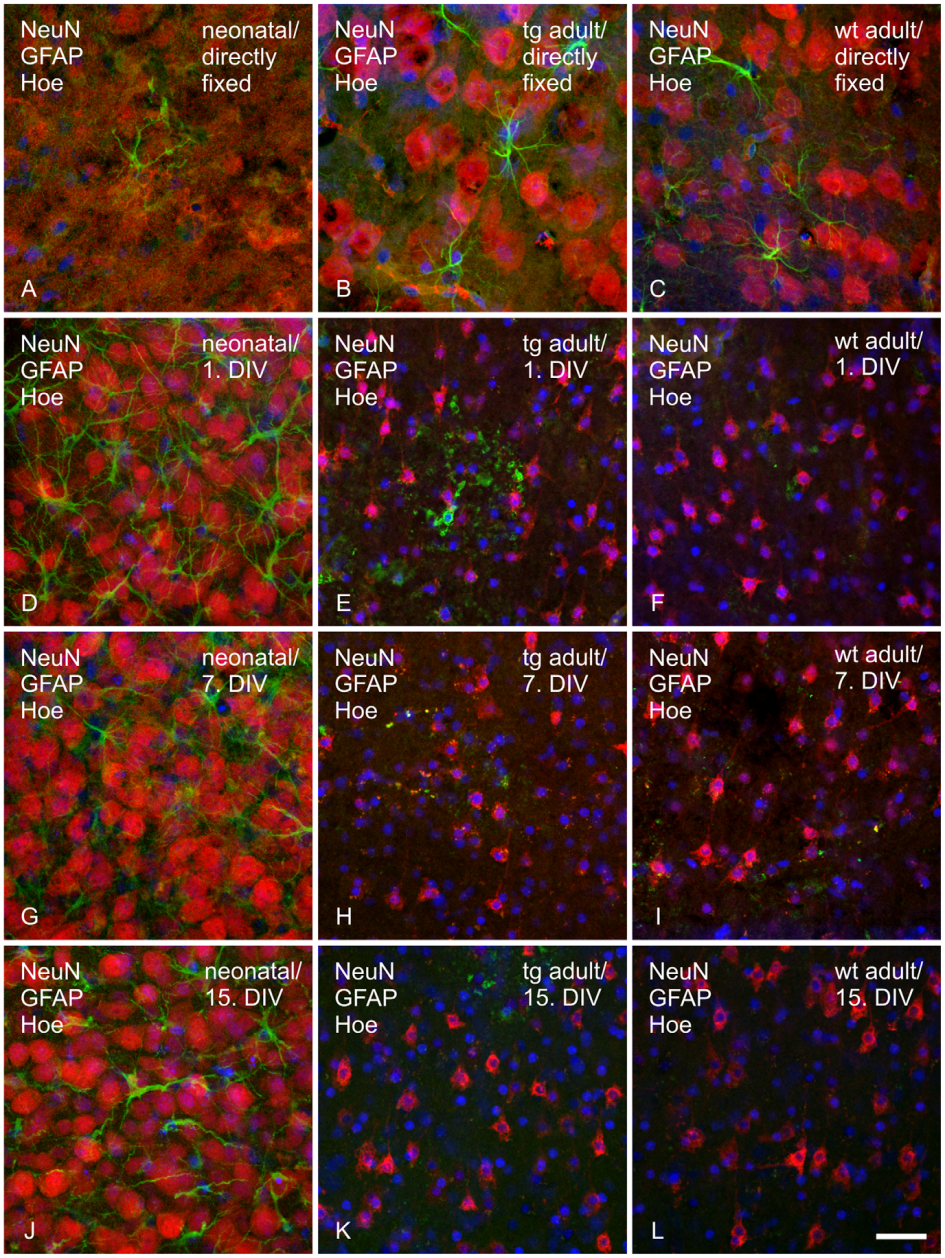
Immunohistochemical labeling of neurons and astrocytes in directly fixed or cultivated brain tissue. Neurons (red), astrocytes (green) and cell nuclei (blue) were visualized with anti-NeuN antibody, anti-GFAP antibody and Hoechst, respectively, in the somatosensory cortex (layer VI) of directly fixed (30 µm) and cultivated brain tissue of neonatal (wt/tg mixed, 300 µm) and adult wild type and transgenic P301S mice (200 µm). A–C: directly fixed tissue; D–F: tissue fixed on 1^st^ DIV; G–I: tissue fixed on 7^th^ DIV; J–L: tissue fixed on 15^th^ DIV. A, D, G, J: neonatal tissue; B, E, H, K: transgenic (tg) adult tissue; C, F, I, L: wild type (wt) adult tissue. Scale bar 20 µm.

#### Nuclear cell morphology

Neonatal nuclear cell morphology was unaffected upon cultivation. The cell nuclei in directly fixed and cultured neonatal tissue showed the same morphology with even staining and obvious small nucleoli (Fig. S4 A, C, E, G). Nuclear appearance in cultivated adult brain tissue was different compared to directly fixed tissue from the 1^st^ DIV to the 15^th^ DIV. The cell nuclei in cultivated adult tissue were more rounded, shrunken and more intensively stained without any obvious nucleoli (Fig. S4 B, D, F, H).

**Figure 4:**
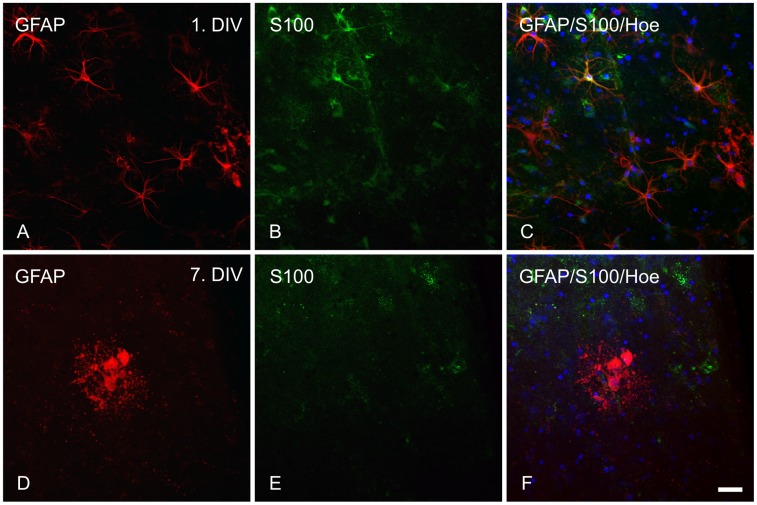
Immunohistochemical labeling of astrocytes (GFAP/S100B) in adult organotypic brain slices. GFAP (red), S100B (green) and cell nuclei (Hoechst, blue) in the somatosensory cortex (layer VI) of cultivated brain slices (200 µm) of adult wild type P301S mice. A–C: tissue fixed on 1^st^ DIV; D–F: tissue fixed on 7^th^ DIV; A, D: GFAP; B, E: S100B; C, F: merged images of GFAP, S100B and Hoechst. Scale bar 20 µm.

#### Neuronal phenotype in cultured neonatal and adult organotypic brain slices

Neurons were visualized with an antibody against NeuN. In neonatal organotypic brain slices neuronal organization and neuronal phenotype retained the form present in directly fixed tissue (Fig. 2 A, D, G, J) without an obvious cortical layering. The cortical layer structure was not visible in the stained wholemount slices, probably due to their thickness. Contrary to that, the typical hippocampal organization was detectable over 15 days of cultivation in adult organotypic brain slices (Fig. S5 A–C). Neuronal density in neonatal cultured slices stayed constant over the cultivation period. In contrast, the number of neurons in cultivated adult slices decreased at the 1^st^ DIV but remained on a constant level afterwards (Fig. 2 B, C, E, F, H, I, K, L). The remaining neurons were found in all cortical layers, but the majority was situated in layer III and VI. Upon cultivation the neurons in cortex and hippocampus changed to an angular shape with shrunken cytoplasm. Neurons lost approximately 2/3 of their cell volume compared to directly fixed tissue. Furthermore, compared to directly fixed tissue spiky and to some extent even longer cell protrusions were visible. No differences between transgenic and wild type adult slices were observed.

**Figure 5:**
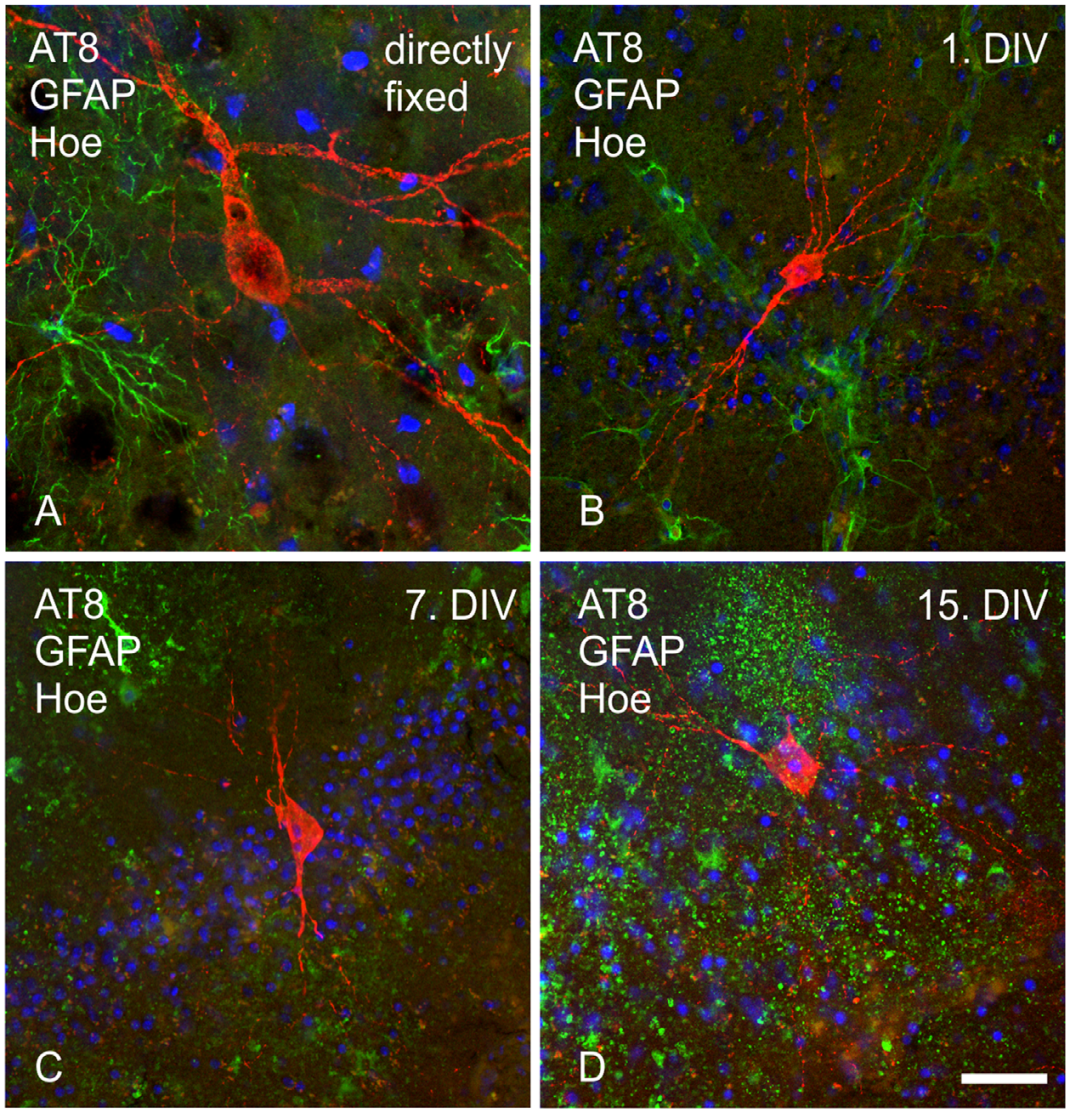
Immunohistochemical labeling of neurofibrillary tangles and astrocytes in P301S transgenic adult organotypic brain slices. Cells positive for hyperphosphorylated human tau (red), astrocytes (green) and cell nuclei were visualized with the antibodies AT8 (anti-human pTau[p202/p205]), anti-GFAP and Hoechst, respectively, in the piriform cortex of directly fixed (30 µm) and cultivated brain tissue (200 µm) of adult transgenic P301S mice. A: directly fixed tissue; B: tissue fixed on 1^st^ DIV; C: tissue fixed on 7^th^ DIV; D: tissue fixed on 15^th^ DIV; Scale bar 20 µm.

#### Microglia phenotype in cultured neonatal and adult organotypic brain slices

Microglial cells were detected with an antibody against the microglia specific protein Iba1 (Fig. 2). Comparable to directly fixed tissue, irregular shaped microglial cells with branched extensions were found from the 1^st^ to the 9^th^ DIV in the cortex of cultivated neonatal slices. From the 11^th^ DIV on, the number of microglial cells in cultivated neonatal slices increased with an even distribution across the cortex (Fig. 2 J; Fig. S6 G, H, I). Microglial cell bodies were swollen on the 1^st^ and 3^rd^ DIV and the extensions were minimized, suggesting an activated cell state (Fig. S6 B, C). From the 5^th^ DIV on, the cell volume decreased again and the branched extensions redeveloped comparable to the ramified *in vivo* cell state (Fig. S6 D). Ramified microglial cells in adult directly fixed tissue revealed long and thin extensions (Fig. 2 B, C). Interestingly, cultivated adult brain slices revealed no Iba1 immunoreactivity over the cultivation period of 15 days (Fig. 2 H, I, K, L). Only on the 1^st^ DIV fully activated, rounded microglial cells without any branches were stained (Fig. 2 E, F). Microglia staining was similar over the entire cultivation period for transgenic and wild type slice cultures.

**Figure 6:**
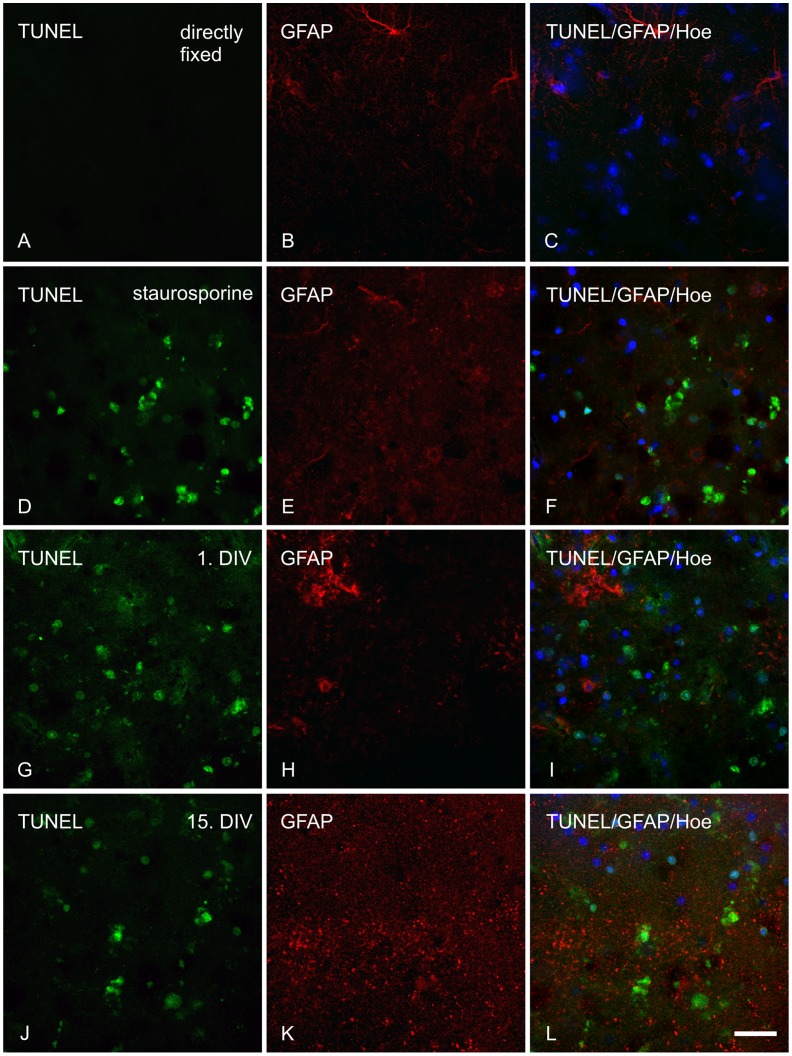
TUNEL staining combined with immunohistochemical labeling of astrocytes in adult organotypic brain slices. Apoptotic cell nuclei (green), astrocytes (red) and cell nuclei (blue) were visualized by TUNEL staining, anti-GFAP and Hoechst, respectively, in the somatosensory cortex (layer VI) of directly fixed (30 µm) and sub-sectioned (30 µm) cultivated brain slices of adult P301S mice (wt/tg mixed). A–C: directly fixed tissue; D–F: tissue treated with staurosporine and fixed on 1^st^ DIV; G–I: tissue fixed on 1^st^ DIV; J–L: tissue fixed on 15^th^ DIV. A, D, G, J: TUNEL; B, E, H, K: GFAP; C, F, I, L: merged images of TUNEL, GFAP and Hoechst. Scale bar 20 µm.

#### Astroglia phenotype in cultured neonatal and adult organotypic brain slices

An antibody against GFAP was used to detect activated astrocytes. Neonatal directly fixed tissue displayed characteristic, star shaped small astrocytes rarely found in the cortical region (Fig. 3 A). In cultivated neonatal slices astrocytes were found in small clusters at the 1^st^ and 3^rd^ DIV (Fig. 3 D, Fig. S7 B, C). After the 5^th^ DIV, the astrocytes were evenly distributed across the whole cortex (Fig. 3 G, J, Fig. S7 D–I) building the typical glial scar. Compared to directly fixed tissue astrocytes in cultivated neonatal slices were hypertrophic with huge cell bodies. In adult cultivated tissue less astrocytes were found compared to directly fixed tissue. Similar to neonatal cultivated slices the cells on 1^st^ DIV were hypertrophic with an increased cell volume (Fig. 3 B, E). After the 3^rd^ DIV to the end of the cultivation period no star shaped astrocytes were apparent. Instead, GFAP-positive round cell bodies surrounded by small dot-like structures were found (Fig. 3 E). A co-staining of GFAP and S100B, a second astrocyte specific protein, revealed a co-localization at the 1^st^ DIV (Fig. 4 C). Surprisingly no co-localization of S100B and GFAP was detected for the dot-like surrounded GFAP positive cells, found after the 1^st^ DIV (Fig. 4 F). Again, the staining of astrocytes and its development over the cultivation period was comparable between transgenic and non transgenic tissue.

#### Tau pathology in cultured adult organotypic brain slices

Tau pathology was detected with the anti-tau antibody AT8, which specifically detects tau phosphorylated at residues Ser202 and Thr205 (numbering according to the longest human isoform with 441 amino acids). Adult wild type slices were not immunoreactive. Single tau-positive cells were localized in the piriform cortex and the hippocampus of transgenic organotypic brain slices and directly fixed tissue. AT8 positive neurons comprised a dense staining of the entire cell body including an axonal distribution without staining of the nucleus (Fig. 5 A) [37]. Contrary to directly fixed tissue, the cell bodies of AT8 positive neurons in cultivated tissue were smaller and more intensively stained without omitting the nucleus (Fig. 5 B, C, D). The size of the stained extensions varied more in cultivated than in directly fixed tissue.

#### TUNEL assay

All analyzed cell types in adult organotypic brain slices morphologically changed towards a shrunken and rounded appearance, suggesting apoptotic events (Fig. 2 and 3, each E, F, H, I, K, L). Apoptotic DNA fragmentation was examined with the TUNEL assay in adult cultivated slices and was present from the 1^st^ DIV up to the 15^th^ DIV (Fig. 6 G, J). These results were also supported by the nuclear morphology present in cultivated adult slices displaying a condensed, pyknotic state already at the 1^st^ DIV. The nuclei did not recover during cultivation and the pyknotic nuclear appearance was found up to the 15^th^ DIV. In contrast, nuclei in neonatal slice cultures did not alter their appearance displaying a healthy nuclear structure typically found in non apoptotic cells (Fig. S2).

## Discussion

### Preparation and Cultivation Conditions

Organotypic brain slice cultures from animals with profound disease related pathology open new insights in biochemical changes and give rise to the possibility to test potential therapeutic substances. Benefits of organotypic brain slices are the good accessibility to native cellular structures, the accustomed interaction of all cells also *ex vivo* and a reduction of the number of animals needed. Interesting results were obtained from rodent neonatal *ex vivo* culture models (e.g. hippocampal brain slice cultures), but these models resemble no suitable tools to model characteristics of age and disease related changes. The age of the applied neonatal rodents is between embryonic state and postnatal day 10 (P10). Only a few studies have tested to cultivate adolescent (P20-P30) [3], [21], [22] or even adult brain tissues (>P50 to 14–16 months) [23], [24], [25], but no systematic optimization about the preparation and cultivation conditions for adult slice cultures has been reported.

This study was designed to evaluate the differences between long term cultures of neonatal mouse brain tissue and brain tissue derived from 7- to 10-month-old tau-transgenic mice. The investigations include the optimization of assays monitoring tissue vitality after various preparation and cultivation conditions and the characterization of the slice cultures with respect to cellular morphology and AD dependent pathology. The vitality of the cultivated slices from one day old neonates and 7- to 10-month-old P301S mice was determined with LDH and MTT assays [27]–[29], whereas propidium iodide labeling of cells with disrupted membranes, as used in other studies, was unfavorable because of high background fluorescence present in non-thinning adult brain tissue (data not shown). For validation of the vitality assays we used the same preparation and cultivation conditions for neonatal and adult brain tissues. The initial parameters adopted from neonatal brain slice preparation and cultivation were: (i) 300 µm slice thickness; (ii) 35°C cultivation temperature; (iii) 5% CO_2_ for cultivation, and (iv) glucose as energy source. As expected, these conditions were suitable for the cultivation of neonatal tissue, but resulted in a 3–8% higher cellular damage and a 50% lower metabolism rate for adult tissue on the 1^st^ DIV. To improve the vitality of the cultivated adult brain tissue various conditions for preparation and cultivation were examined (tables 1 and 2). Among the tested settings the thickness of the slices, the cultivation temperature and CO_2_ concentration had the most significant effects with respect to vitality. The thickness of neonatal slices decreased from 300–400 µm to approximately 100–150 µm after 1 to 2 week of incubation [17], while the mature adult slices nearly kept their thickness over a two week cultivation period. As oxygen diffuses only around 100 µm into brain tissue at 35°C, as shown in guinea pig brain slices [38], the central cellular layers of adult slices died probably upon oxygen starvation. After reduction of the slice thickness from 300–400 µm to 200 µm the cell death was minimized as shown by a 30–50% increased metabolism rate. The pH of the cultivation media was maintained by CO_2_ fumigation. The difference between the incubator CO_2_ concentration (5%) and the atmospheric CO_2_ concentration (<0.05%) results in a lethal alkaline pH shift as found for neuronal cell cultures [31]. To minimize the pH variations caused by the low atmospheric CO_2_ concentration during media exchange every other day, the incubator CO_2_ concentration was reduced to 2.5%. However, the decreased CO_2_ concentration deteriorated the vitality of our adult organotypic brain slices, probably due to metabolic differences between primary single cell culture and whole tissue slices. The cultivation temperature for adolescent organotypic brain slices ranges from 37°C [24], [39] to mild hypothermic conditions at 34°C [3], [22], [23], [34], [40] and moderate hypothermic conditions at 32°C [21], [41] or even extreme hypothermia at 30°C [42]. In organotypic brain slice models used for ischemia [29] and traumatic brain injury [43], moderate hypothermia decreased neuronal death. Additionally, a long hypothermic phase was found to be beneficial for slices concerning long-term functionality and morphology [44], [45]. The preparation process of brain slices damages the tissue and almost certainly causes ischemic circumstances. It has been shown that moderate hypothermic cultivation conditions at 32°C caused a higher necrosis rate compared to mild hypothermic conditions at 35°C for adult organotypic brain slices. The higher LDH efflux is probably caused by “fragile” neuronal cell membranes as described for slices being stored below 33°C [46]. Furthermore, intra-ischemic hypothermia was found to be even more neuroprotective than post-ischemic hypothermia [47], [48]. However, it was demonstrated that per cardiac perfusion with ice-cold preparation buffer before brain excision had no influence on the slice vitality compared to direct brain preparation.

### Metabolic Influences

In the present study, no significant changes in the vitality levels of adult brain slices related with changes in the composition of the cultivation medium were observed. The standard energy source for brain tissue is glucose, but brain cells are also able to metabolize lactate [49], [50] and ketone bodies like β-hydroxybutyrate [51], [52]. A medium containing all three energy substrates (glucose, lactate, β-hydroxybutyrate) did not improve the vitality of our adult slices. Normal horse serum was added to the medium for supplementation with e.g. cytokines and hormones. But the composition of the sera varies strongly with the batch number and has had a severe influence on the vitality of adolescent cultures [41]. Exchanging the serum with the standardized B27 supplement increased the LDH efflux. Therefore B27 supplement seems not to be sufficient for the cultivation of mature tissue, indicating that additional factors present in normal horse serum might be important for cell survival in adult slice cultures. An increased potassium concentration was applied in several studies using neonatal or adolescent tissue for brain slice preparation inducing neuronal depolarization and elevated rates of spontaneous activity [21], [33], [40]. In the present study a potassium concentration of 10 mM induced no significant vitality improvement for adult organotypic brain slices suggesting that the neurons in adult brain slices lost their capacity for being depolarized. Survival promoting peptides originated from a mouse hippocampal cell line have been proven to increase the vitality of organotypic brain slice cultures [53]. As already mentioned above, ischemic conditions are likely to occur during the preparation process, but the application of survival promoting peptides did not influence the slice vitality. A reason for the missing positive effect of the survival promoting peptides could be found in different tolerances against oxygen glucose deprivation between neonatal and adult organotypic brain slices [41]. The same was true for the ionotrophic NMDA receptor antagonist MK-801 and the antioxidant butylated hydroxytoluene. MK-801 reduced glutamate dependent toxicity in adolescent hippocampal slices of rat brain, but was not sufficient to support their long-term cultivation [23]. In contrast, no vitality improving effect of MK-801 was found in a similar experiment for adolescent hippocampal rat brain slices [42], which is comparable to our study on adult organotypic murine brain slices. Similar effects were described for butylated hydroxytoluene, which is thought to reduce the TNFα induced glutamate toxicity [35]. Here no protective influence of MK-801 and butylated hydroxytoluene were found for adult organotypic brain slices, suggesting that glutamate toxicity may not to be the main apoptosis inducer. To further address the issue of possible apoptosis initiation, we also included an antisense oligodesoxynucleotide to knock-down the connexin 43 expression [34], [54]. This should reduce toxic signaling between neurons at the slice surfaces through gap junctions [34], although for adult organotypic brain slices no vitality increase following knock-down of connexin 43 was illustrated. This could be caused by the fact that the knock-down additionally hampers astrocyte communication, which has an essential role in tissue regeneration.

### Changes in Morphology

The vitality of the major cell populations, namely neurons, astrocytes and microglia, in the neonatal and adult organotypic brain slice cultures, was determined by immunohistochemistry characterizing their morphology and activation state over 15 days. Perfusion fixed brain tissue was applied as control in this study, because it resembles most precisely the *in vivo* situation. The comparison between perfusion fixed *in vivo* slices and cultivated slices *ex vivo* points out all differences caused by preparation and cultivation. The structural changes of the glial cells in neonatal organotypic brain slices were comparable to alterations observed in other studies. Through the preparation process activated microglial cells returned to a resting state after the 3^rd^ DIV [55], [56] and the typical glial scar consisting of hypertrophic astrocytes was found from the 5^th^ DIV on [57]. Neuronal appearance and the entire nuclear structure in neonatal organotypic brain slices were not altered during the cultivation period. In contrast, the morphology of all three analyzed cell types changed dramatically in adult organotypic brain slices, reflecting their decreased vitality level. All neurons displayed a severe loss of cell volume with a non-vital angular cell shape. Also the cell nuclei transformed to a shrunken, pyknotic state. In conjunction with the high amount of TUNEL-positive cell nuclei, these results are indicative for apoptotic changes. We observed a massive neuronal cell loss in all cortical areas of adult organotypic brain slices with the exception of layers III and VI. Interestingly, an elevated cell death, especially in these layers (II/III and VI), is described for neonatal cultivated tissue (P4) [33]. This is different to the situation *in vivo*, where cell death arises in layer II rather than in layers IV to VI in the first postnatal week [58]–[61]. The activation of microglial cells is always accompanied by neuronal damage [62]. The observed microglial cell activation in the present study was only detected on the 1^st^ DIV in adult organotypic brain slices. Furthermore overactivation, which causes microglia to perish [63], is possibly a fundamental self-regulatory mechanism to protect neurons from bystander damage and might also be seen in our adult brain slice cultures. Possible microglia overactivation following the preparation negatively affects neuronal vitality ending with microglial cell apoptosis, as Iba1-positive microglial cells were only found on the 1^st^ DIV. Microglial cells in the presented adult brain slices omit the possibility to return to a resting state after activation as microglial cells in neonatal slices do [56]. Another indication for the complete removal of microglia in adult slices was the absence of the cell elimination processes by which necrotic cells are usually removed within 2–3 days in neonatal slices [33]. Different to the microglia occurrence, GFAP-positive cells were found over the entire cultivation period of 15 days, but only on the 1^st^ DIV the cells resembled their typical star shaped structure. After the 3^rd^ DIV instead, small GFAP-positive cells with dot-like structures around the cell body were detected missing the characteristic extensions of astrocytes. This appearance is described in a process called clasmatodendrosis as a result of an irreversible injury of astrocytes after ischemia [64], [65]. Astrocytes undergoing clasmatodendrosis are in a preapoptotic state without the ability to perceive their tissue protective function. Different to microglia, LPS treatment does not lead to overactivation of astrocytes with following apoptosis [63]. Our results support this notion, as after potential overactivation of glia by the preparation process, microglial cells were only found at the 1^st^ DIV but astrocytes over the whole cultivation period. Additional to GFAP, several astrocytes express the S100B protein. Low concentrations (nM) of S100B revealed a trophic function for neurons whereas high concentrations (µM) showed deleterious effects [66]. S100B was found to activate microglia [67] which is always accompanied with neuronal damage [62]. We only observed S100B positive astrocytes on the 1^st^ DIV. In conjunction with the missing microglia we assume that neurons are negatively affected through both, astrocytes and microglia, in the adult organotypic brain slices.

Supplementary to neurons and glial cells we examined the present tau pathology over the cultivation period. AD associated AT8-positive neurons were visible over the whole cultivation period, but the appearance of cells changed slightly. Under the used *ex vivo* conditions, these tau positive neurons are embedded in a more deleterious environment than under the situation for AD *in vivo*, where simply activated microglia and astrocytes are present. Surprisingly, no accelerated neuronal cell loss was observed in slices from transgenic adult animals compared to slices from wild type adult animals, an additional hallmark of AD pathology. The order of cell death is different to AD, with glial cells dying before neurons in cultivated adult slices, which is most likely caused by ischemic damage and not by the transgenic modification of the tissue. In contrast to these findings, cultivated primary neurons are more vulnerable to ischemic damage compared to cultivated astrocytes [68], [69]. Otherwise, *in vivo* glial cells are sometimes found to be damaged earlier than neurons [70]–[72].

In summary, the presented data illustrate the metabolic and morphologic variance between neonatal and mature adult organotypic brain slice cultures. The slow processing cell death observed in the adult brain slice model is unfavorable to study age-related or Alzheimer’s disease-related mechanisms, but may serve as a potential model system to study neuroprotection, as suggested in other studies [39], [73].

## Materials and Methods

### Ethics Statement

The study was approved by the local authorities (Landesdirektion Leipzig, license number T113/10) following the guidelines of the German Animal Welfare Act. All efforts were made to reduce the number of animals and to minimize animal suffering. Adult animals were deeply anesthetized with isofluran followed by decapitation. Neonatal mice (P1) were directly decapitated.

### Animal Model

P301S mice [26] were purchased from Jackson Laboratory (Bar Harbor, MA, USA) and where housed at 12 h light/dark cycles with unlimited excess to water and food. The transgene construct contains the P301S mutant of the human microtubule-associated protein tau (MAPT) under the direction of the mouse prion protein (Prnp) promoter. The expression of the mutant human MAPT is 5-fold higher than the expression of the endogenous mouse tau protein. The transgene construct was injected into fertilized B6C3F1 mouse ova.

### Organotypic Brain Slice Preparation

Brain tissue was obtained from 1-day and 7- to 10-month-old P301S heterozygous transgenic and wild type mice. Neonatal animals were directly decapitated, whereas adult animals were anesthetized with isofluran (Baxter, Unterschleißheim, Germany) prior to decapitation. The brain was removed and immediately immersed in ice-cold Ringer solution (2.5 mM KCl (Sigma-Aldrich, Steinheim, Germany), 1 mM MgCl_2_ (Sigma-Aldrich), 260 mM D-Glucose (Carl Roth GmbH, Karlsruhe, Germany), 26 mM NaHCO_3_ (Sigma-Aldrich), 1.25 NaH_2_PO_4_ (Sigma-Aldrich), 2 mM pyruvic acid (Sigma-Aldrich), 3 mM myo-inositol (Sigma-Aldrich), 1 mM kynuric acid (Merck, Darmstadt, Germany), 2 mM CaCl_2_ (Sigma-Aldrich), pH 7.3 for two minutes. The cerebellum was trimmed off and the caudal end of the brain was glued onto the cutting table of the vibratome (7000 smz Campden instruments). The brain was cut in coronal slices of 200–400 µm with an amplitude of 1.5 mm, a frequency of 75 Hz and a velocity of 0.1 mm/s. The slices were collected and stored in ice-cold Ringer solution before floating onto semi-porous membrane inserts (Millipore, 0.4 µm pore diameter, Schwalbach, Germany). Damaged slices were discarded. Intact slices were cultivated at 35°C and 5% CO_2_ in a standard medium consisting of DMEM Ham’s F12 (pH 7.3) and 24% normal horse serum, antibiotic mixture (5 µg/mL penicillin, 5 µg/mL streptomycin and 10 µg/mL neomycin) (all from Invitrogen, Karlsruhe, Germany) and additional 10 mM D-glucose (Sigma-Aldrich). Medium was changed every other day. As control for the immunohistochemical analysis of the organotypic brain slices, directly fixed brain tissue was used as described as follows. Age matched control mice were deeply anesthetized with carbon dioxide and transcardially perfused with PBS (4 mM, pH 7.4) followed by perfusion with 4% paraformaldehyde in PBS. Brains were removed, postfixed for 16 h at 4°C and stored in 30% sucrose in PBS (0.4 mM, pH 7.4). Coronal sections (30 µm) were cut from frozen brain tissue on a cryostat.

### Alternative Preparation and Cultivation Conditions

To increase the vitality of adult organotypic brain slice cultures the preparation and cultivation conditions were modified as follows: I) Transcardial perfusion with 10 mL ice-cold Ringer solution. II) Slice thicknesses: 200 µm, 300 µm, 400 µm. III) Cultivation temperatures: 32°C and 35°C. IV) Cultivation CO_2_ concentrations: 2.5% and 5%. V) Substrate mixture medium consisted of standard medium with 2 mM lactate (Sigma-Aldrich) and 2 mM β-hydroxybutyrate (Sigma-Aldrich) and was applied directly after preparation. B27 medium (DMEM Ham’s F12 with standard antibiotic mixture, 10 mM D-glucose and B27 supplement (Invitrogen)) was used from the 3^rd^ DIV on. The slices were cultivated in standard medium for the first two days. VI) High potassium concentration: 10 mM. VII) Survival promoting peptides (SPPs) were synthesized and purified in house and used as acetic acid salt at 200 nM in standard medium. SPP1: YDPEAASAPGSGNPCHEASAAQCENAGEDP, SPP2: DPEAASAPGSGNPCHEA and SPP3: CHECSAAQC. VIII) Survival promoting additives: MK-801 (30 µM added to Ringer solution, Sigma-Aldrich); once right after preparation 2 µM connexin 43 antisense oligodesoxynucleotide (Metabion, Planegg-Martinsried, Germany) in 70 µL pluronic F-127 gel (Sigma-Aldrich) on top of each slice for 6 h in B27 medium (afterwards standard medium); butylated hydroxytoluene (300 µM, Sigma-Aldrich) added to the standard medium over the whole cultivation period.

### Vitality Assays

The LDH assay was used to quantify the LDH release, which correlates to the number of necrotic cells. The assay medium was collected every other day and frozen at −20°C. On the 15^th^ DIV the slices were harvested together with the medium, sonicated to extract the remaining LDH (350 J over 2 min with 2 s pulse and 3 s pause) and centrifuged (13000g, 4°C, 30 min). The LDH concentration in the supernatant was quantified in a 96-well format. Therefore, 25 µL sample were mixed with 175 µL 0.1 M phosphate buffer, pH 7.5 containing 10 µmol/mL sodium pyruvate (Sigma-Aldrich) and 100 nmol/mL NADH (Sigma-Aldrich). NADH decrease was monitored at 340 nm over 2 min. To calculate the percentage of released LDH the enzyme activity was divided by the slice volume (volume of elliptical cylinder  =  (slice length/2)*(slice width/2)*slice thickness). The MTT assay is based on the conversion of the yellow tetrazolium salt MTT to purple formazan crystals by metabolic active cells. MTT (stock solution 5 mg/mL in PBS, pH 7.4) was added to the cultivation media to a final concentration of 0.5 mg/mL. The slices were incubated for four hours under cultivation conditions, washed with PBS, and transferred into a mixture of SDS (10%, w/v, Roth), DMF (25%, v/v, Biosolve, Valkenswaard, Netherlands) and water to dissolve the formazan crystals. After 24 hours the samples were centrifuged and the absorbance of the supernatant (150 µL) was recorded at 562 nm and 690 nm. The metabolic activity was calculated as follows: (A_562_– A_690_)/slice volume. In both assays triton X-100 (1%) was used as a control for cell death after four hours of incubation. Each analysis was performed in duplicate with 3 to 4 slices per time point for the MTT assay and 5 to 6 wells (each containing 2 slices) per time point for the LDH assay.

### Immunohistochemistry

For immunohistochemical analysis the brain tissue was fixed with 4% paraformaldehyde (Sigma-Aldrich) in PBS (4 mM, pH 7.4, Invitrogen) for 16 h at 4°C followed by three times washing for 15 min in TBS (50 mM, pH 7.4). Slice cultures were stored in TBS. Wholemount sections without any reslicing and directly fixed control slices were blocked for 1 h with 5% fetal calf serum (FCS, Invitrogen) in TBS containing 0.3% triton X-100. Primary antibodies specific for astrocytes, e.g. glial fibrillary acidic protein (rabbit anti-GFAP, 1∶500, DAKO, Hamburg, Germany) and S100B protein (rabbit anti-S100B, 1∶200; abcam, Cambridge, United Kingdom); microglia specific Iba1 (rabbit anti-Iba1, 1∶100, Wako, Neuss, Germany), neuron specific protein (mouse anti-NeuN, 1∶100, Millipore) or human phosphorylated tau protein (mouse anti-Tau, clone AT8, 1∶500, Thermo Scientific, Schwerte, Germany) were applied in blocking solution. The slices were incubated overnight at 4°C with orbital shaking, washed three times with TBS (5 min each), and incubated with species specific fluorescence-labeled secondary antibodies (Cy3 donkey anti-mouse, 1∶800, Cy2 donkey anti-rabbit, 1∶400; both Jackson ImmunoResearch, West Grove, USA) in blocking solution for four hours. The cell nuclei were stained subsequently with Hoechst (1 µg/mL; Invitrogen) for 10 min in blocking solution. The procedure was finalized with three washing steps before the tissue slices were mounted on glass slides. The slides were dried over night, dehydrated twice with acetic acid butyl ester (Roth, 5 min) before embedding in entellan (Merck, Darmstadt, Germany). Control experiments were performed without primary antibodies.

Images were taken at a confocal laser scanning microscope (LSM 510 Meta, Zeiss) using excitation wavelengths of 543 nm (helium/neon1, red Cy3-fluorescence), 488 nm (argon, green Cy2-fluorescence) and 351, 364 nm (UV, blue Hoechst fluorescence).

### TUNEL-Assay

Control slices were treated with 5 µM staurosporine (Roche) for 16 h [74]. Fixation for immunohistochemical analysis was done with 4% paraformaldehyde in PBS (4 mM, pH 7.4) for 16 h at 4°C followed by cryoprotection in 30% sucrose (Sigma-Aldrich) dissolved in diluted PBS (0.4 mM, pH 7.4). Slices were frozen in 30% sucrose and sub-sectioned on a cryostat at a thickness of 30 µm. The TUNEL assay (Roche, Mannheim, Germany) was conducted according to manufacturer’s protocol and combined with immunohistochemical labeling. Briefly, 550 µL buffer (reagent A) were mixed with 50 µL enzyme solution (reagent B). Each slice was incubated with 100 µL of this solution at 37°C for 1 h protected by a coverslip. Immunohistochemical staining was conducted afterwards as described above.

### Statistical Analysis

Statistical analysis was performed with Graph Pad Prism (version 3.02) first testing parametric distribution followed by Students-t-test (two parameter) and ANOVA (three parameter) including Bonferroni correction. Means ± standard deviation are given throughout.

## Supporting Information

Figure S1 **Graphical representation of cellular metabolism and necrosis of cultivated transgenic and wild type brain tissue.** A: MTT assay was applied to compare cellular metabolism between wild type and transgenic organotypic brain slices. B: LDH assay was used to compare cellular necrosis between wild type and transgenic organotypic brain slices.
(TIF)

Figure S2 **Immunohistochemical labeling of neurons and astrocytes in directly fixed and cultivated brain tissue.** Neurons (red), astrocytes (green) and cell nuclei (blue) were visualized with anti-NeuN antibody, anti-Iba1 antibody and Hoechst, respectively, in the somatosensory cortex (layer VI) of directly fixed (30 µm) and cultivated brain tissue of neonatal (wt/tg mixed, 300 µm) and adult wild type and transgenic P301S mice (200 µm). A–C: directly fixed; D–F: fixed on 1^st^ DIV; G–I: fixed on 7^th^ DIV; J–L: fixed on 15^th^ DIV. A, D, G, J: neonatal tissue; B, E, H, K: transgenic (tg) adult tissue; C, F, I, L: wild type (wt) adult tissue. Scale bar 50 µm.
(TIF)

Figure S3 **Immunohistochemical labeling of neurons and microglia in directly fixed and cultivated brain tissue.** Neurons (red), microglia (green) and cell nuclei (blue) were visualized with anti-NeuN antibody, anti-Iba1 antibody and Hoechst, respectively, in the somatosensory cortex (layer VI) of directly fixed (30 µm) and cultivated brain tissue of neonatal (wt/tg mixed, 300 µm) and adult wild type and transgenic P301S mice (200 µm). A–C: directly fixed; D–F: fixed on 1st DIV; G–I: fixed on 7th DIV; J–L: fixed on 15th DIV. A, D, G, J: neonatal tissue; B, E, H, K: transgenic (tg) adult tissue; C, F, I, L: wild type (wt) adult tissue; G, J: neonatal tissue; B, E, H, K: transgenic (tg) adult tissue; C, F, I, L: wild type (wt) adult tissue. Scale bar 50 µm.
(TIF)

Figure S4 **Comparison of nuclear cell staining with Hoechst in neonatal and adult organotypic brain slices.** Cell nuclei (Hoechst) are shown in blue. A, B: directly fixed tissue; C, D: tissue fixed on 1^st^ DIV; E, F: tissue fixed on 7^th^ DIV; G, H: tissue fixed on 15^th^ DIV. A, C, E, G: neonatal tissue; B, D, F, H transgenic adult tissue. Images taken in somatosensory cortex, layer VI. Slice thickness was 30 µm for directly fixed tissue, 300 µm for cultivated neonatal tissue and 200 µm for cultivated adult tissue. Scale bar 10 µm.
(TIF)

Figure S5 **Immunohistochemical labeling of neurons and astrocytes in the hippocampus of cultivated adult brain tissue.** Neurons (red), astrocytes (green) and cell nuclei (blue) were visualized with anti-NeuN antibody, anti-GFAP antibody and Hoechst, respectively, in the hippocampus of cultivated brain tissue (200 µm) of adult wildtyp P301S mice. A: fixed on 1^st^ DIV; B: fixed on 7^th^ DIV; C: fixed on 15^th^ DIV. Scale bar 100 µm.
(TIF)

Figure S6 **Immunohistochemical labeling of microglia in directly fixed and cultivated neonatal brain tissue of P301S mice.** A: directly fixed tissue (30 µm); B–I: fixed after cultivation as indicated (300 µm). Scale bar 50 µm.
(TIF)

Figure S7 **Immunohistochemical labeling of astrocytes in directly fixed and cultivated neonatal brain tissue of P301S mice.** A: directly fixed tissue (30 µm); B–I: fixed after cultivation as indicated (300 µm). Scale bar 50 µm.
(TIF)
