# Supplementary figures and images for: Organotypic Brain Slice Cultures of Adult Transgenic P301S Mice—A Model for Tauopathy Studies
Source: PLoS One. 2012 Sep 11;7(9):e45017. doi: 10.1371/journal.pone.0045017 (PMC3439393; doi:10.1371/journal.pone.0045017)

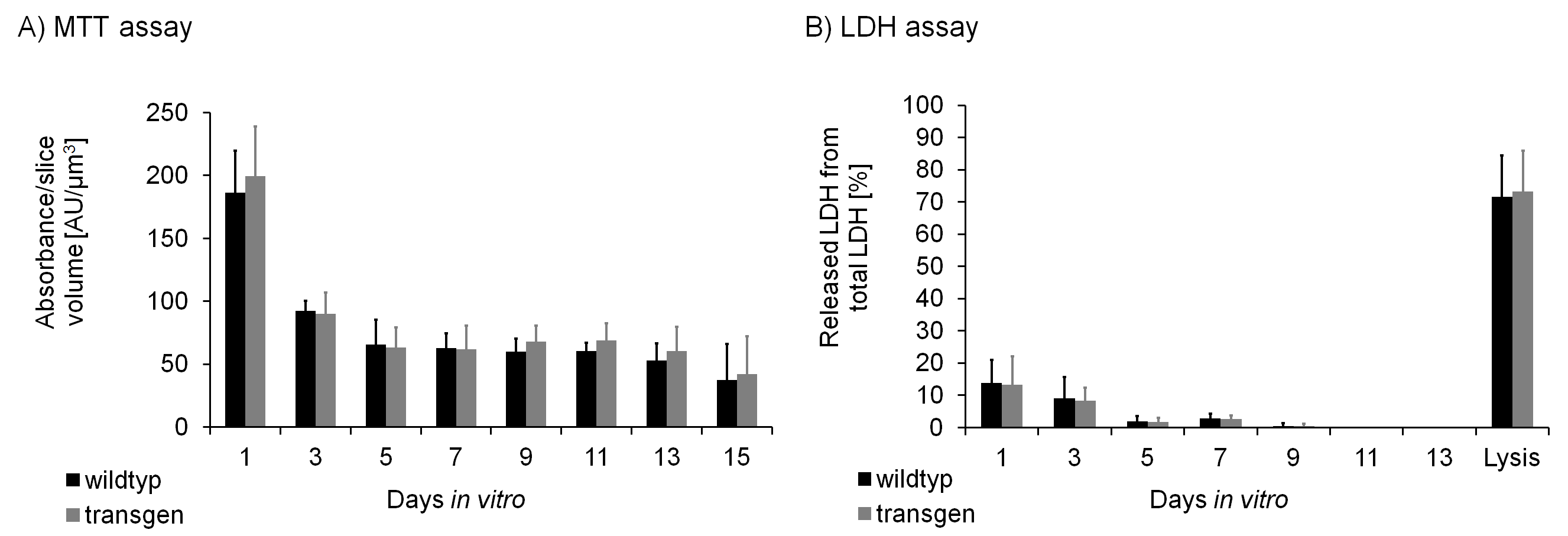

Supplement: Figure S1 — Graphical representation of cellular metabolism and necrosis of cultivated transgenic and wild type brain tissue. A: MTT assay was applied to compare cellular metabolism between wild type and transgenic organotypic brain slices. B: LDH assay was used to compare cellular necrosis between wild type and transgenic organotypic brain slices. (TIF) [file pone.0045017.s001.tif]

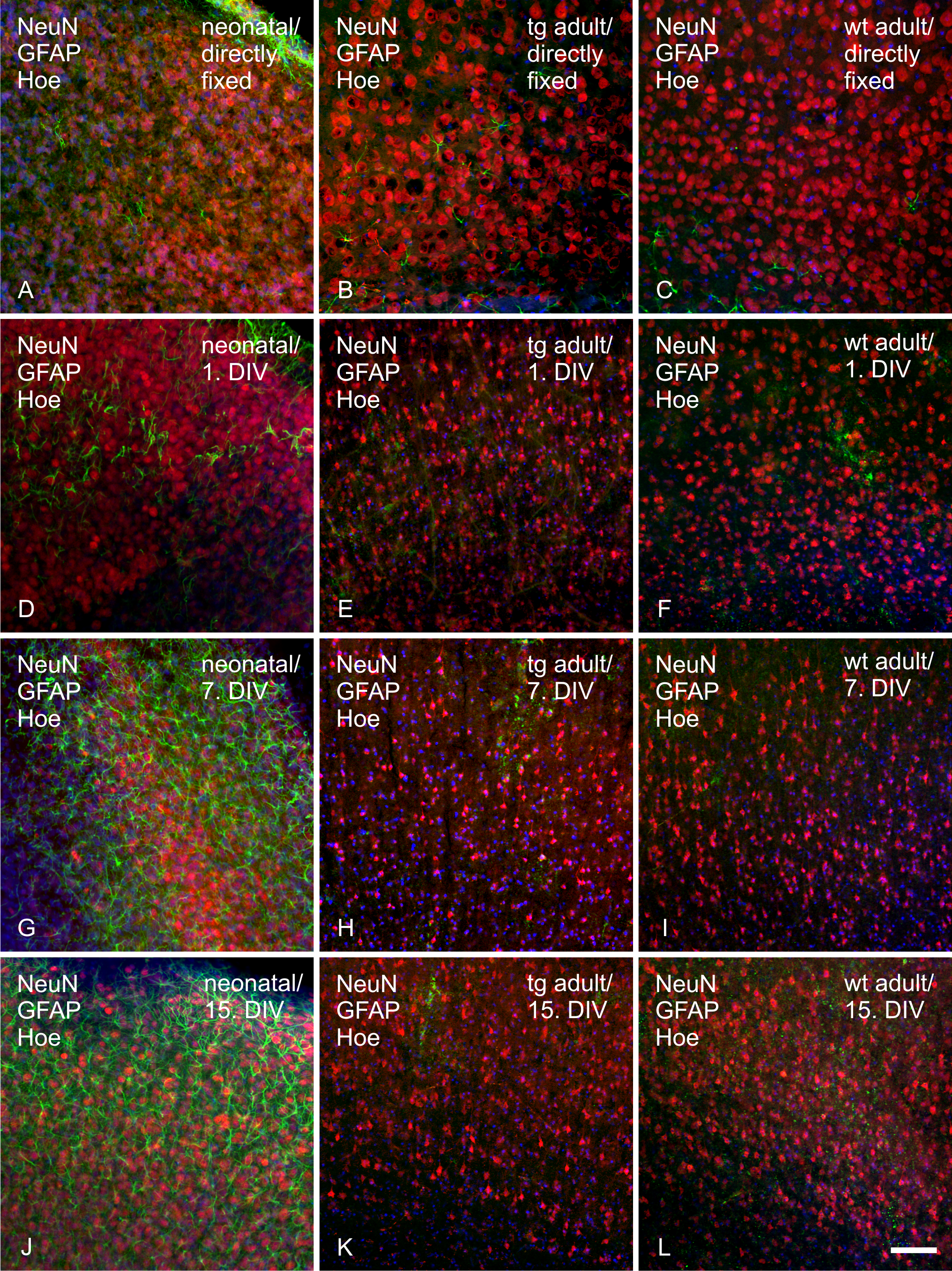

Supplement: Figure S2 — Immunohistochemical labeling of neurons and astrocytes in directly fixed and cultivated brain tissue. Neurons (red), astrocytes (green) and cell nuclei (blue) were visualized with anti-NeuN antibody, anti-Iba1 antibody and Hoechst, respectively, in the somatosensory cortex (layer VI) of directly fixed (30 µm) and cultivated brain tissue of neonatal (wt/tg mixed, 300 µm) and adult wild type and transgenic P301S mice (200 µm). A–C: directly fixed; D–F: fixed on 1st DIV; G–I: fixed on 7th DIV; J–L: fixed on 15th DIV. A, D, G, J: neonatal tissue; B, E, H, K: transgenic (tg) adult tissue; C, F, I, L: wild type (wt) adult tissue. Scale bar 50 µm. (TIF) [file pone.0045017.s002.tif]

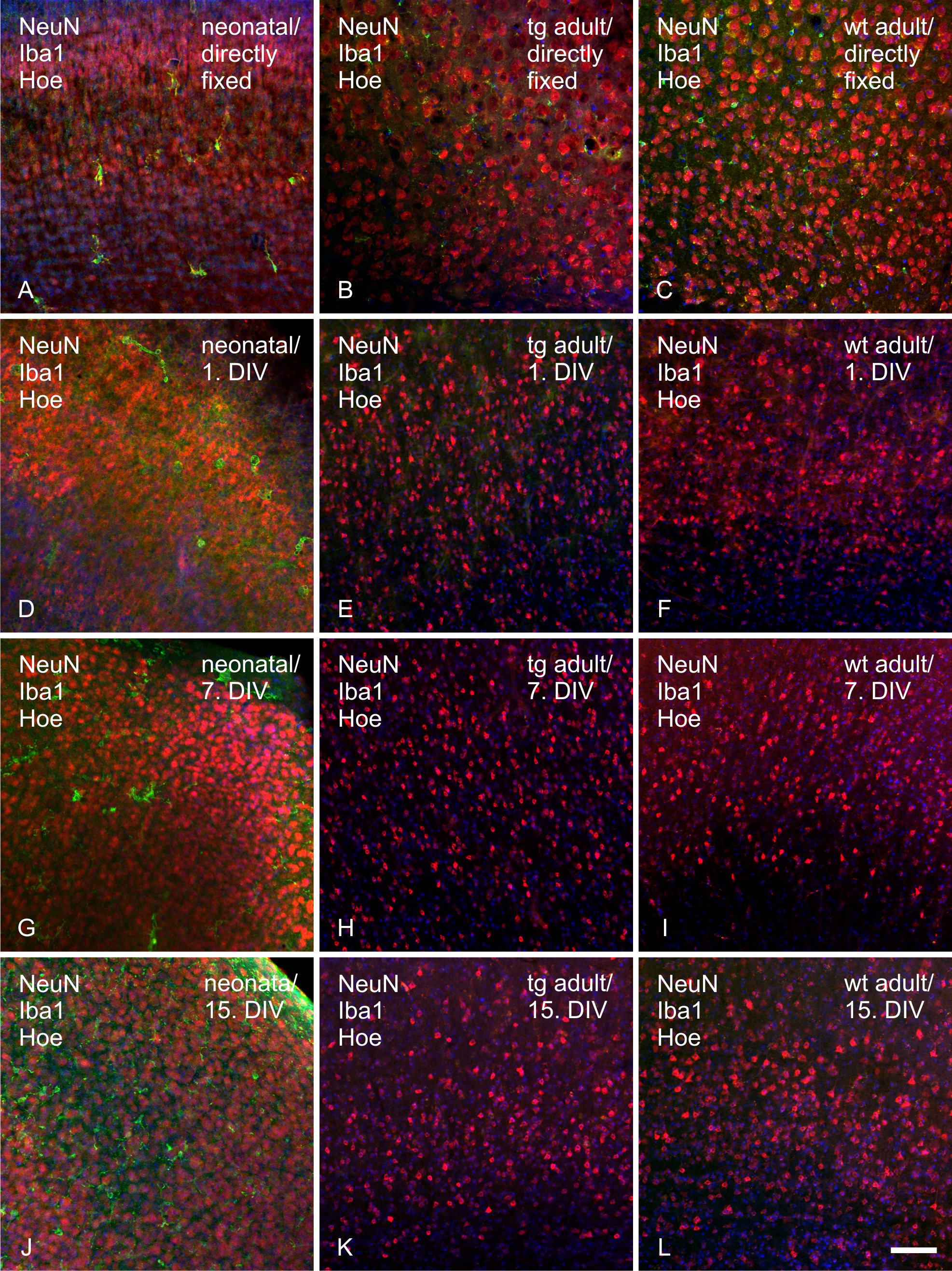

Supplement: Figure S3 — Immunohistochemical labeling of neurons and microglia in directly fixed and cultivated brain tissue. Neurons (red), microglia (green) and cell nuclei (blue) were visualized with anti-NeuN antibody, anti-Iba1 antibody and Hoechst, respectively, in the somatosensory cortex (layer VI) of directly fixed (30 µm) and cultivated brain tissue of neonatal (wt/tg mixed, 300 µm) and adult wild type and transgenic P301S mice (200 µm). A–C: directly fixed; D–F: fixed on 1st DIV; G–I: fixed on 7th DIV; J–L: fixed on 15th DIV. A, D, G, J: neonatal tissue; B, E, H, K: transgenic (tg) adult tissue; C, F, I, L: wild type (wt) adult tissue; G, J: neonatal tissue; B, E, H, K: transgenic (tg) adult tissue; C, F, I, L: wild type (wt) adult tissue. Scale bar 50 µm. (TIF) [file pone.0045017.s003.tif]

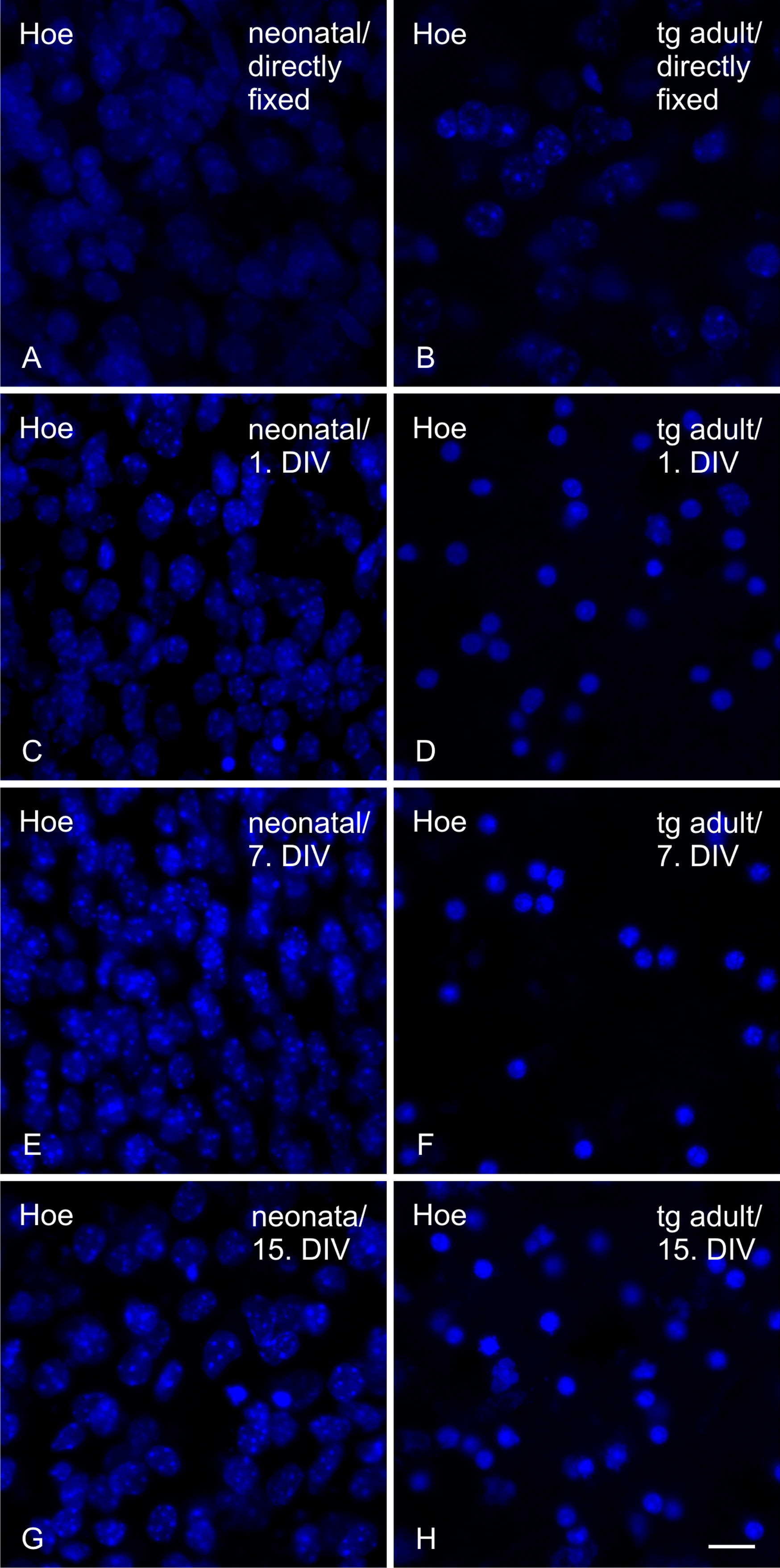

Supplement: Figure S4 — Comparison of nuclear cell staining with Hoechst in neonatal and adult organotypic brain slices. Cell nuclei (Hoechst) are shown in blue. A, B: directly fixed tissue; C, D: tissue fixed on 1st DIV; E, F: tissue fixed on 7th DIV; G, H: tissue fixed on 15th DIV. A, C, E, G: neonatal tissue; B, D, F, H transgenic adult tissue. Images taken in somatosensory cortex, layer VI. Slice thickness was 30 µm for directly fixed tissue, 300 µm for cultivated neonatal tissue and 200 µm for cultivated adult tissue. Scale bar 10 µm. (TIF) [file pone.0045017.s004.tif]

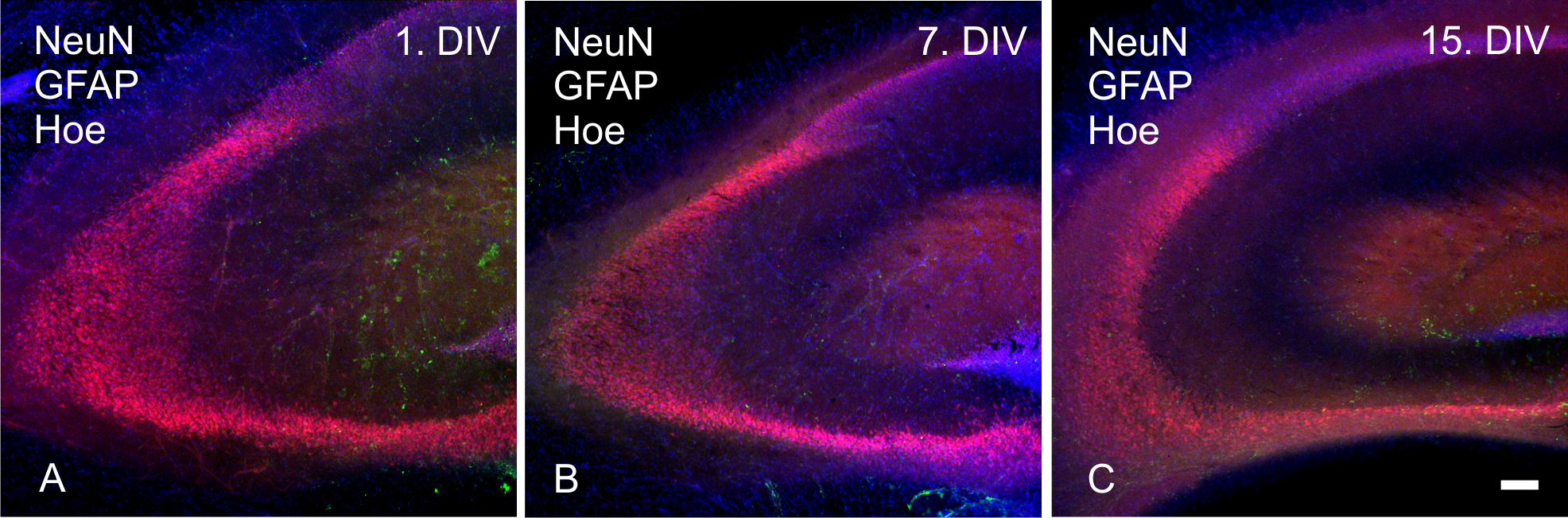

Supplement: Figure S5 — Immunohistochemical labeling of neurons and astrocytes in the hippocampus of cultivated adult brain tissue. Neurons (red), astrocytes (green) and cell nuclei (blue) were visualized with anti-NeuN antibody, anti-GFAP antibody and Hoechst, respectively, in the hippocampus of cultivated brain tissue (200 µm) of adult wildtyp P301S mice. A: fixed on 1st DIV; B: fixed on 7th DIV; C: fixed on 15th DIV. Scale bar 100 µm. (TIF) [file pone.0045017.s005.tif]

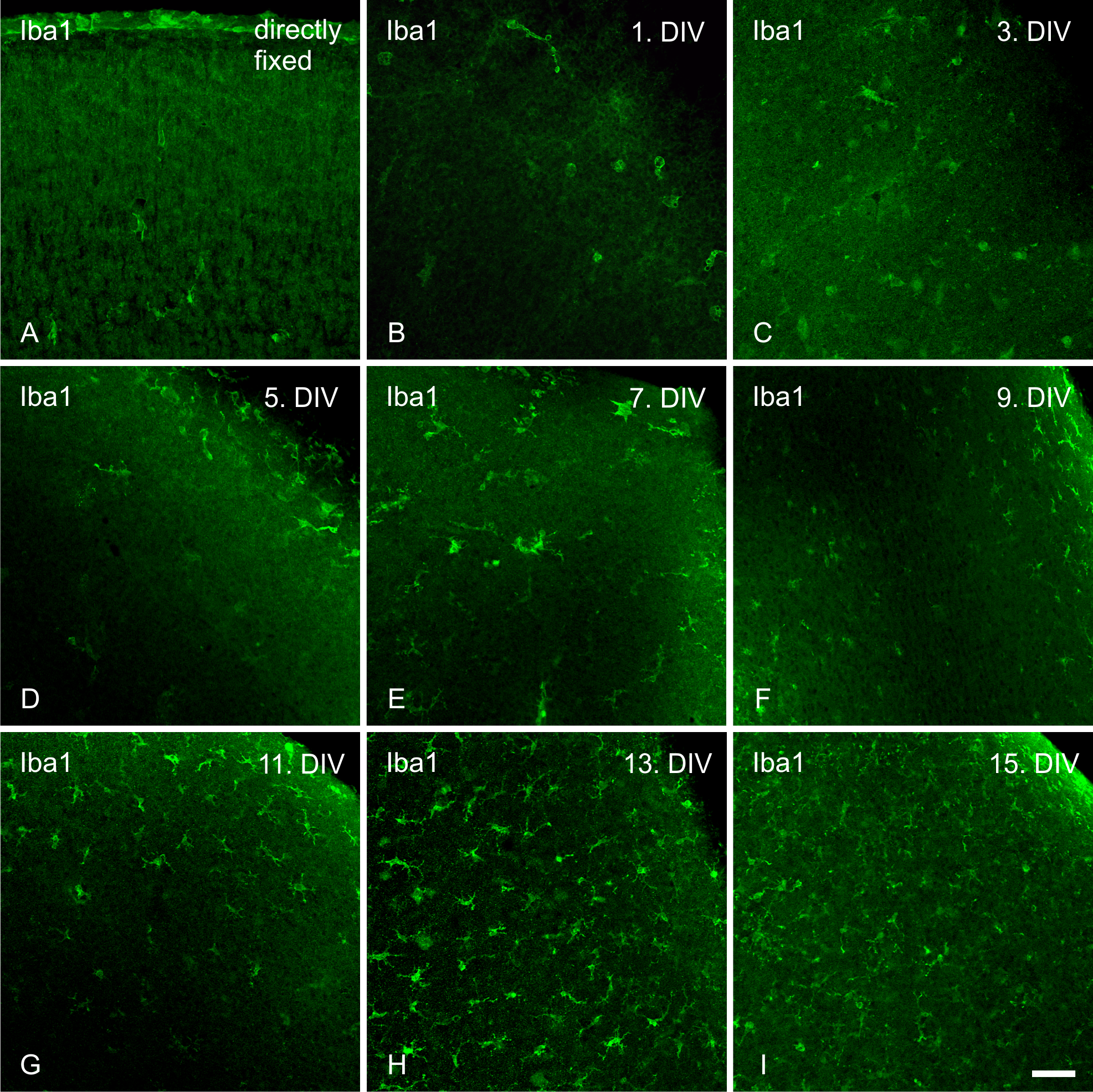

Supplement: Figure S6 — Immunohistochemical labeling of microglia in directly fixed and cultivated neonatal brain tissue of P301S mice. A: directly fixed tissue (30 µm); B–I: fixed after cultivation as indicated (300 µm). Scale bar 50 µm. (TIF) [file pone.0045017.s006.tif]

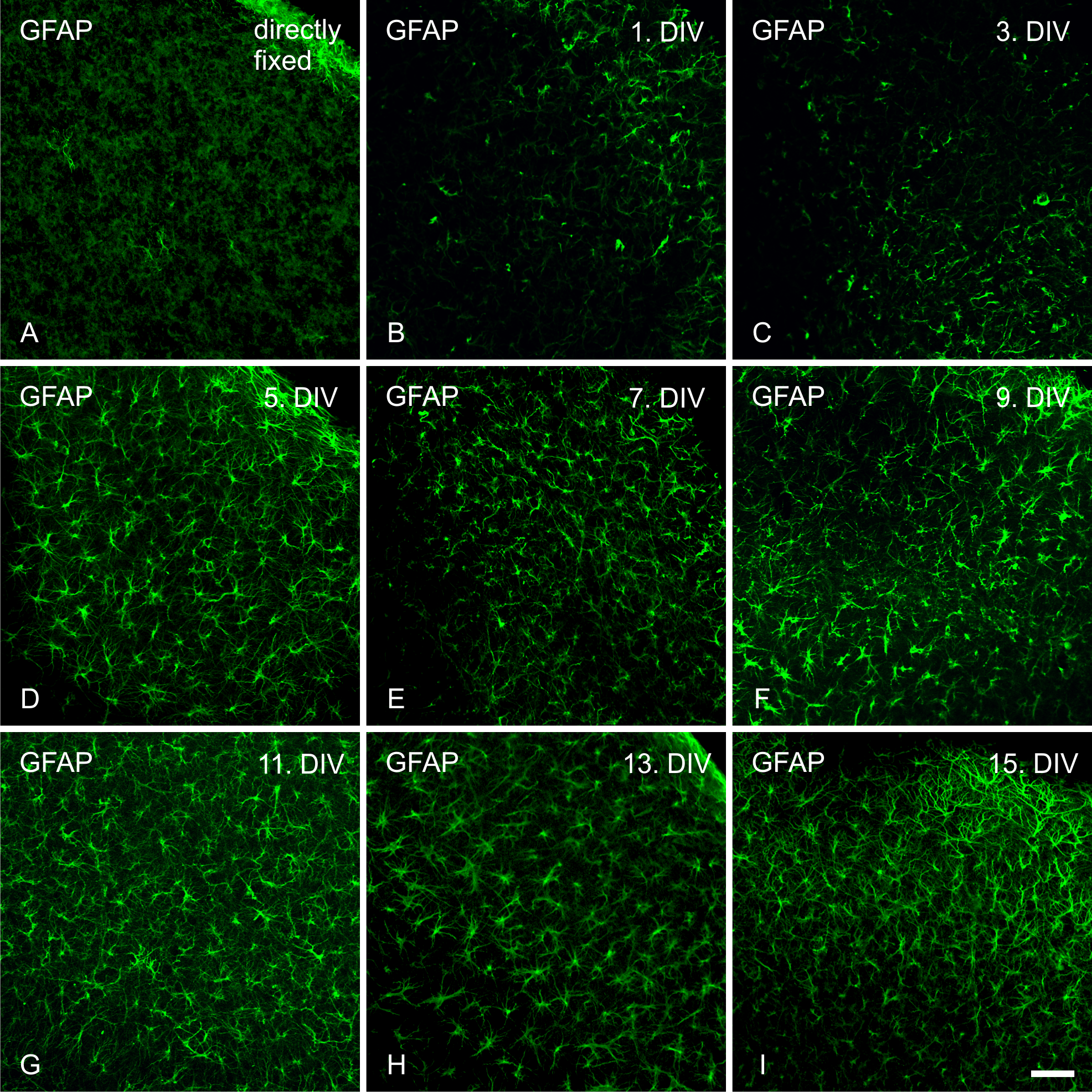

Supplement: Figure S7 — Immunohistochemical labeling of astrocytes in directly fixed and cultivated neonatal brain tissue of P301S mice. A: directly fixed tissue (30 µm); B–I: fixed after cultivation as indicated (300 µm). Scale bar 50 µm. (TIF) [file pone.0045017.s007.tif]
